# Supplementary material for: Antigen persistence and TLR stimulation contribute to induction of a durable HIV-1-specific neutralizing antibody response
Source: Nat Commun. 2025 Jun 3;16:5162. doi: 10.1038/s41467-025-60481-2 (PMC12134134; doi:10.1038/s41467-025-60481-2)
Supplement: Supplementary file 1 — Supplementary Information [file 41467_2025_60481_MOESM1_ESM.pdf]

Antigen Persistence and TLR-Stimulation Contribute to Induction of a Durable HIV-1-Specific Neutralizing Antibody Response

Kenta Matsuda[1], Mitra Harrison[1], Eleanor Wettstein[1], Jessica Pederson[1], Alyssa A. Pullano[1], Lyuba Bolkhovitinov[1], Breanna Kim[1], Isabel Steinberg[1], Trevor Griesman[1], Sarah Stuccio[1], Daniel Rogan[1], Andy Patamawenu[1], Tulley Shofner[1], Nathaniel E. Wright[1], Jonathan D. Webber[1], Freya van't Veer[1], Rachel Roenicke[1], Emma Koory[1], Peyton M. Roeder[1], Ellison Ober[1], Benjamin Leach[1], Yaroslav Tsybovsky[2], Tyler Stephens[2], Ivan Del Moral-Sanchez[3], Ilja Bontjer[3], Lori W. McGinnes-Cullen[4], Eric Chu[5], Jason Liang[5], Jonathan L. Torres[6], Ryan N. Lin[6], Andy S. Tran[6], Gabrielle Dziubla[7], Leonid Serebryannyy[7], Sandeep Narpala[7], Bob Lin[7], Mike Castro[7], Gabriel Ozorowski[6], Andrew B. Ward[6], Rogier W. Sanders[3], Peter D. Kwong[7], Javier Guenaga[8], Richard Wyatt[8], Trudy Morrison[4] and Mark Connors[1]*.

*To whom correspondence should be addressed
mconnors@nih.gov

[1]HIV-Specific Immunity Section of the Laboratory of Immunoregulation, National Institute of Allergy and Infectious Diseases, National Institutes of Health, Bethesda, MD 20892, USA.
[2]Electron Microscopy Laboratory, Cancer Research Technology Program, Leidos Biomedical Research Inc., Frederick National Laboratory for Cancer Research, Frederick, MD 21701, USA.
[3]Department of Medical Microbiology and Infection Prevention, Amsterdam University Medical Centers, Location AMC, University of Amsterdam, Amsterdam, the Netherlands.
[4]Department of Microbiology and Physiological Systems, Sherman Center, University of Massachusetts Medical School, Worcester, MA 01655, USA.
[5]Division Of Clinical Research, Biostatistics Research Branch, National Institute of Allergy and Infectious Diseases, National Institutes of Health, Bethesda, MD, USA
[6]Department of Integrative Structural and Computational Biology, The Scripps Research Institute, La Jolla, CA 92037, USA.
[7]Vaccine Research Center, National Institute of Allergy and Infectious Diseases, National Institutes of Health, Bethesda, MD 20892, USA.
[8]International AIDS Vaccine Initiative Neutralizing Antibody Center, Department of Immunology and Microbiology, The Scripps Research Institute, San Diego, CA 92037, USA

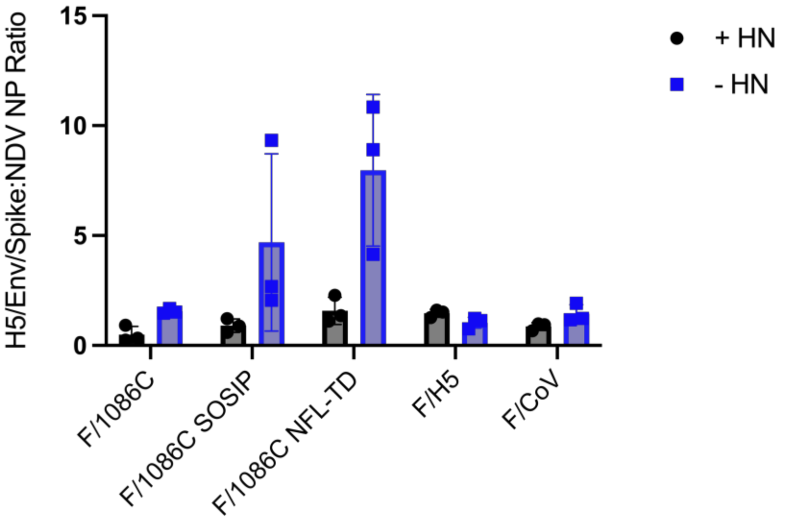

Supplementary Figure 1. Optimization of chimeric F protein incorporation to VLP.
The ratio of chimeric NDV F/Env, H5, spike proteins to NDV nucleoprotein measured by Western blot. Transient transfection was performed with or without inclusion of plasmid expressing NDV HN protein to produce VLPs (n=3). The samples derive from the same experiment and the blots were processed in parallel.

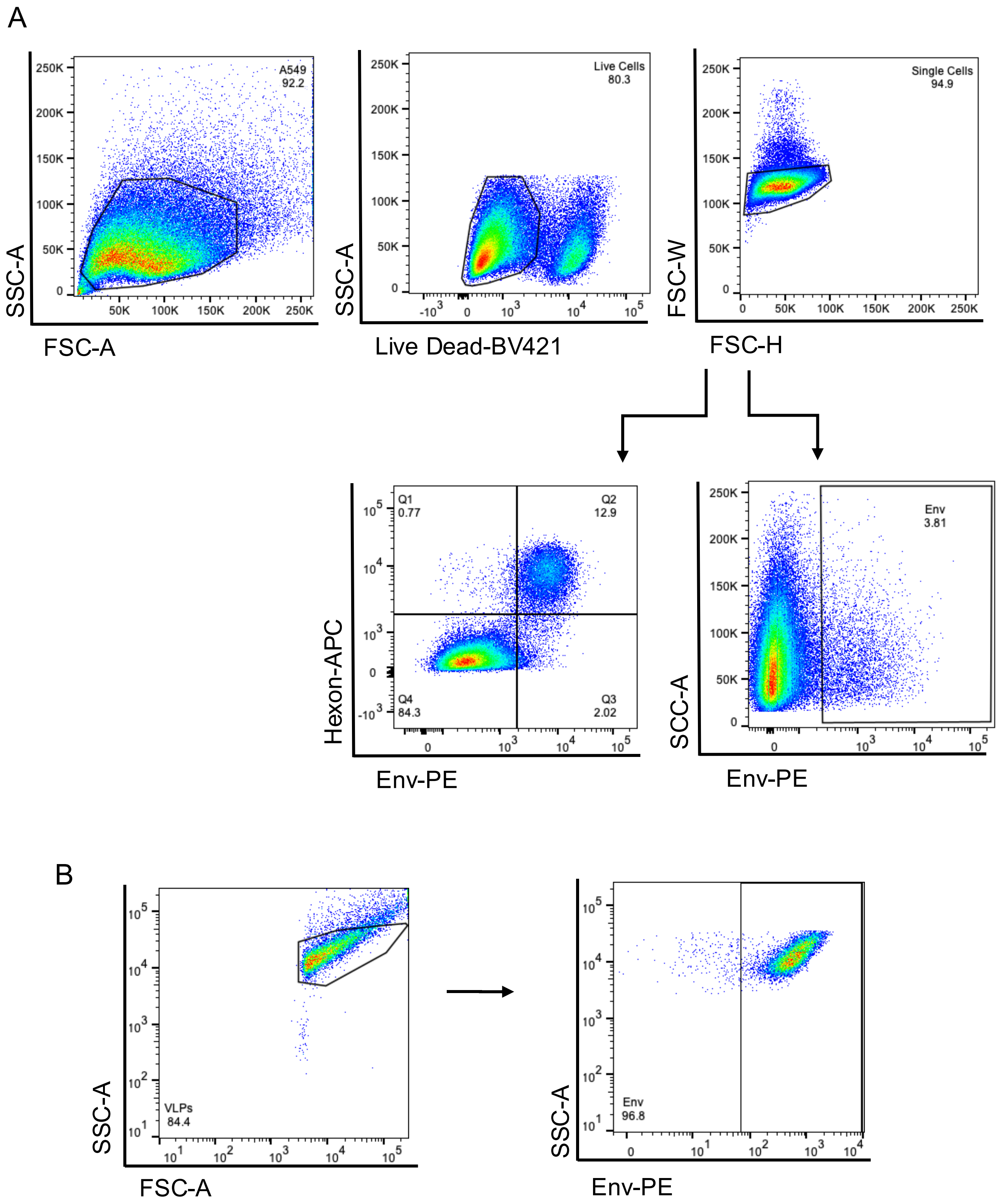

Supplementary Figure 2. Flow Cytometry and virometry gating strategy

(A) Surface staining of Env on A549 cells transfected with NDV plasmids and infected with Ad4-Env.

(B) The presence of HIV-1 Env antigens on the surface of VLPs was determined by flow virometry.

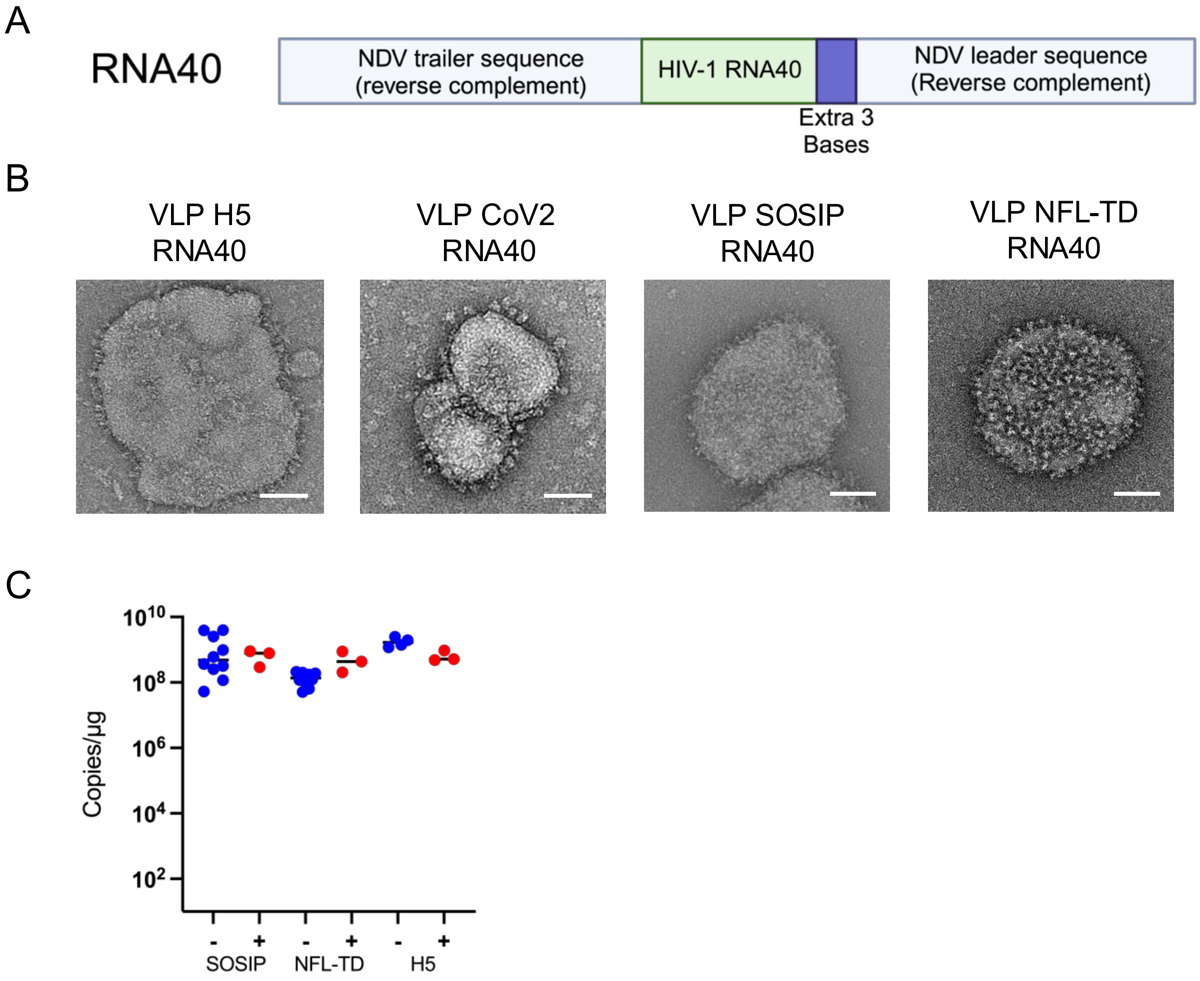

Supplementary Figure 3. Incorporation of TLR agonist RNA40 into NDV VLP.

(A) Schematic of the expression plasmid containing the RNA40 cDNA sequence with flanking NDV leader and trailer sequences.

(B) Representative EM images of VLP variants expressing RNA40. The scale bars correspond to 50nm.

(C) Detection of RNA40 transcripts incorporated to NDV VLP. qPCR was performed on VLPs treated with/without RNase treatment during purification step (SOSIP and NFL-TD w/o RNase n=10, H5 w/o RNase n=4; SOSIP, NFL-TD, and H5 with RNase n=3).

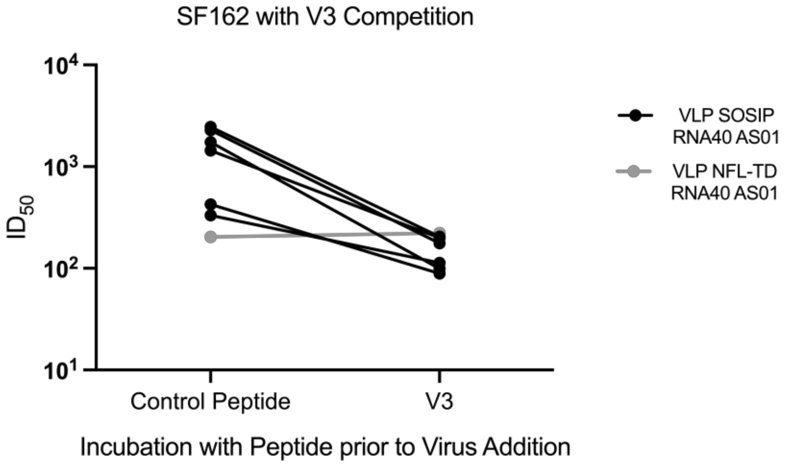

Supplementary Figure 4. V3 Competition assay

V3 or control peptide was co-incubated with serum prior to assaying SF162 pseudovirus neutralization. V3 peptide significantly reduced neutralization of sera from animals immunized with VLP SOSIP RNA40 using an escalating dose (SOSIP p-value: **0.0146) (n=8).

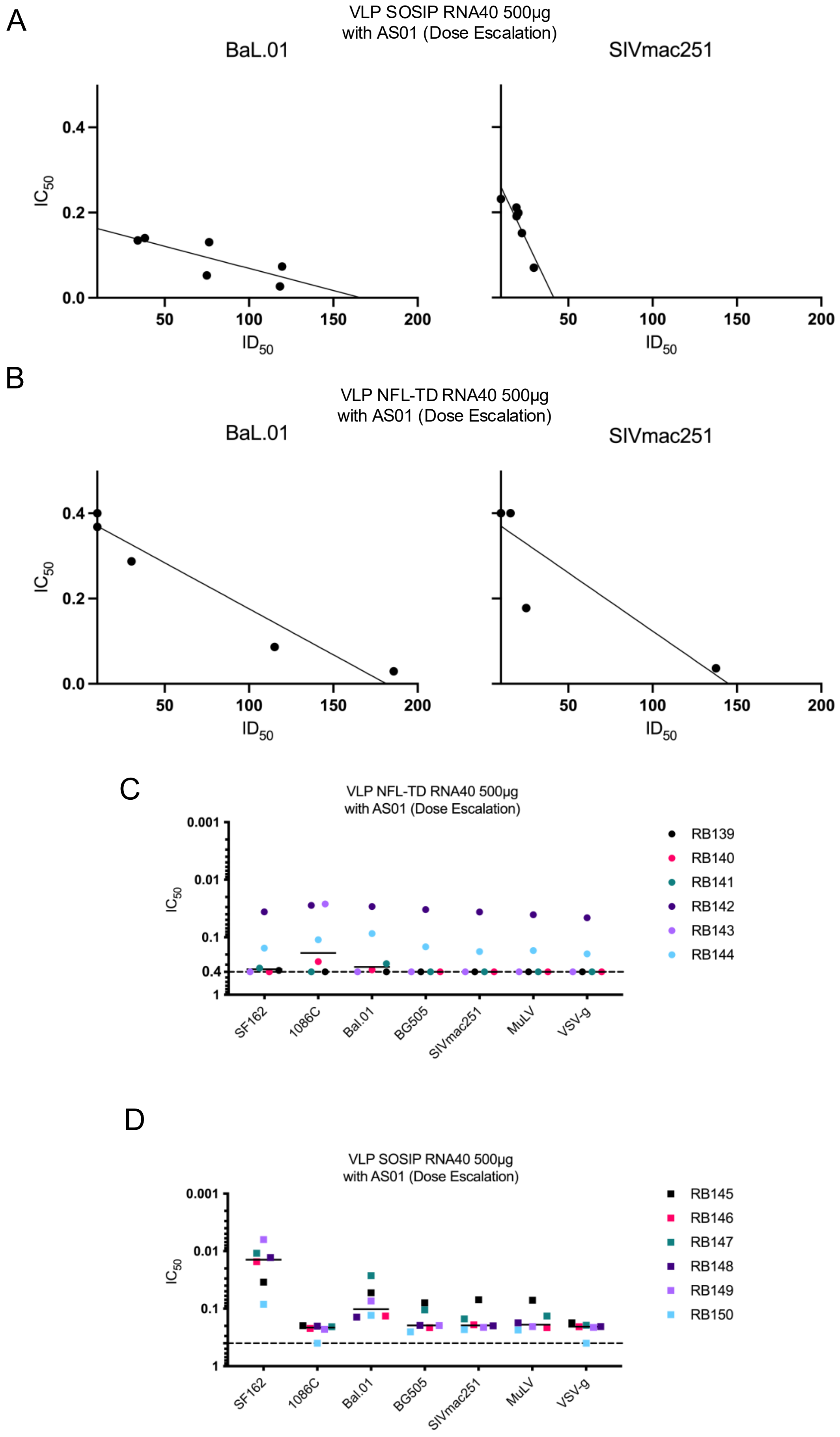

Supplementary Figure 5. Purified Ig neutralization of HIV and non-HIV pseudoviruses
(A, B) Inverse correlation of IC_{50} of purified Ig with the ID_{50} of serum for the same animal (n=6).
(C, D) Neutralizing activity of Ig purified from sera of rabbits that received the indicated vaccine via dose escalation against a panel of four HIV and three non-HIV pseudoviruses (n=6). Median IC_{50} titers are indicated.

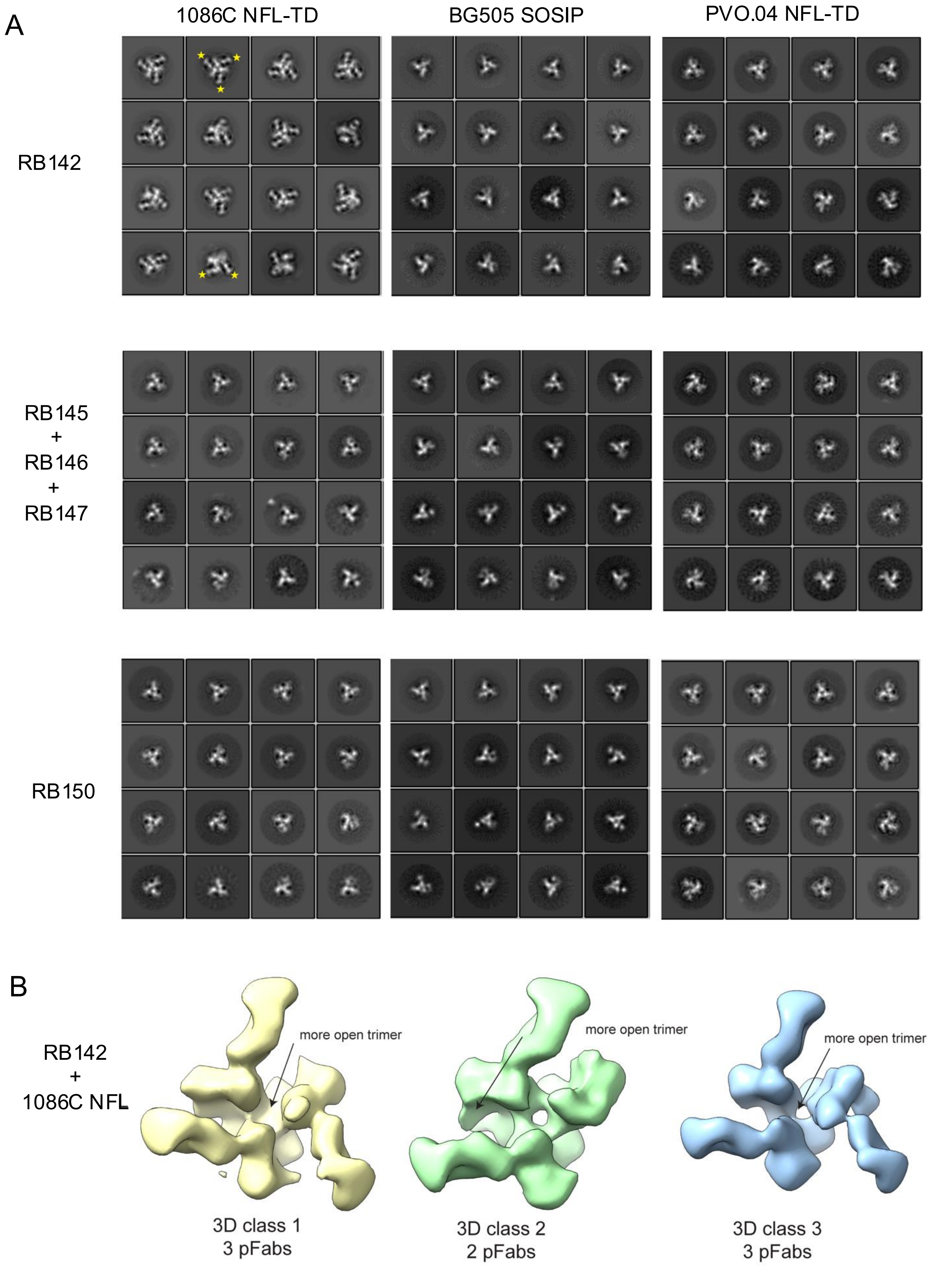

Supplementary Figure 6. Electron microscopy-based polyclonal epitope mapping (EMPEM) of select immunized rabbits.

(A) Representative 2D classes of 1086C NFL, BG505 SOSIP or PVO.04 NFL trimers incubated with week 43 polyclonal Fab (pFab) from rabbits RB142, RB150 or pooled samples from RB145, RB146 and RB147. Fab-bound trimers are visible in the RB142 pFab + 1086C NFL sample (top left), with representative pFab densities marked with a star. Fab-bound trimers were not detected in all other pFab-trimer complexes tested.

(B) Representative 3D classes of RB142 week 43 pFab + 1086C NFL reveal stoichiometries of 2 or 3 Fabs per trimer, with greater trimer opening (gap between gp120 subunits) at the apex.
