# Supplementary material for: Antigen persistence and TLR stimulation contribute to induction of a durable HIV-1-specific neutralizing antibody response
Source: Nat Commun. 2025 Jun 3;16:5162. doi: 10.1038/s41467-025-60481-2 (PMC12134134; doi:10.1038/s41467-025-60481-2)
Supplement: Supplementary file 2 — Reporting Summary [file 41467_2025_60481_MOESM2_ESM.pdf]

nature portfolio

Corresponding author(s): Mark Connors

Last updated by author(s): Apr 24, 2025

Reporting Summary

Nature Portfolio wishes to improve the reproducibility of the work that we publish. This form provides structure for consistency and transparency in reporting. For further information on Nature Portfolio policies, see our Editorial Policies and the Editorial Policy Checklist.

Statistics

For all statistical analyses, confirm that the following items are present in the figure legend, table legend, main text, or Methods section.

n/a	Confirmed	
☐	☒	The exact sample size (*n*) for each experimental group/condition, given as a discrete number and unit of measurement
☐	☒	A statement on whether measurements were taken from distinct samples or whether the same sample was measured repeatedly
☐	☒	The statistical test(s) used AND whether they are one- or two-sided<br>*Only common tests should be described solely by name; describe more complex techniques in the Methods section.*
☐	☒	A description of all covariates tested
☐	☒	A description of any assumptions or corrections, such as tests of normality and adjustment for multiple comparisons
☐	☒	A full description of the statistical parameters including central tendency (e.g. means) or other basic estimates (e.g. regression coefficient) AND variation (e.g. standard deviation) or associated estimates of uncertainty (e.g. confidence intervals)
☐	☒	For null hypothesis testing, the test statistic (e.g. *F*, *t*, *r*) with confidence intervals, effect sizes, degrees of freedom and *P* value noted<br>*Give P values as exact values whenever suitable.*
☒	☐	For Bayesian analysis, information on the choice of priors and Markov chain Monte Carlo settings
☒	☐	For hierarchical and complex designs, identification of the appropriate level for tests and full reporting of outcomes
☒	☐	Estimates of effect sizes (e.g. Cohen's *d*, Pearson's *r*), indicating how they were calculated

Our web collection on statistics for biologists contains articles on many of the points above.

Software and code

Policy information about availability of computer code

Data collection	-Flow cytometry data was collected with BD FACSDiva software v.10.9.0 (Becton Dickinson, Franklin Lakes, NJ, USA)<br>-Western blot analysis were collected using the ChemiDoc MP Imaging system (Bio-Rad Laboratories, Hercules, CA, USA).<br>-qPCR data were collected using QuantStudio 3/5 Real-Time PCR software and Thermo Fisher Connect Platform (Thermo Fisher Scientific, Waltham, MA ,USA).<br>-Mesoscale Discovery Assay dataset was collected using the MSD Sector Imager S600 (Meso Scale Discovery, Rockville, MD, USA).<br>-Serum neutralization and competitive inhibitions assay datasets were collected using the Perkin Elmer 2030 Workstation software (Perkin Elmer, Waltham, MA, USA).<br>-Electron microscopy-based polyclonal epitope mapping (EMPEM) data was collected using a FEI Tecnai Spirit T12 equipped with an FEI Eagle 4k x 4k CCD camera (120 keV, 2.06 Å/pixel) or a FEI Thermo Fisher Scientific Glacios equipped with a Thermo Fisher Scientific Falcon IV direct electron detector (200 keV, 1.89 Å/pixel).<br>-Negative stain electron microscopy data was acquired using a Talos F200C transmission electron microscope that was equipped with a Ceta CCD camera.
Data analysis	-FlowJo software v.10.10.0 was used for flow cytometry data analysis.<br>-ImageJ (Version 2.1.0/1.53c) was used for Western blot analysis.<br>-Graph Pad Prism version 10.2.0 (335) was used for plotting graphs of serum neutralization datasets and analyzing the Mesoscale Discovery Assay.<br>-qPCR QuantStudio 3/5 Real-Time PCR Software and Thermo Fisher Connect Platform was used to analyze the RNA40 transcript expression levels in VLPs.<br>-Serum neutralization and competitive inhibition data were analyzed on Microsoft Excel version 16.95.4.<br>-EMPEM data was processed using Relion 3.0 and composite maps generated using UCSF Chimera.

-Negatvie stain electron microscopy data was analyzed using Spider and Relion 3.0.
-R version version 4.4.0 was used for statistical analysis

For manuscripts utilizing custom algorithms or software that are central to the research but not yet described in published literature, software must be made available to editors and reviewers. We strongly encourage code deposition in a community repository (e.g. GitHub). See the Nature Portfolio guidelines for submitting code & software for further information.

Data

Policy information about availability of data

All manuscripts must include a data availability statement. This statement should provide the following information, where applicable:

- Accession codes, unique identifiers, or web links for publicly available datasets
- A description of any restrictions on data availability
- For clinical datasets or third party data, please ensure that the statement adheres to our policy

All data supporting the findings in this study are available within the article and its Supplementary Information. The source data underlying Figs. 1-7, Supplementary Figs. 1-6 and Supplementary Tables 1 and 2 are provided as a Source Data file. The negative-stain EMPEM map has been deposited to the Electron Microscopy Data Bank under accession code EMD-49656 (https://www.ebi.ac.uk/emdb/EMD-49656).

Research involving human participants, their data, or biological material

Policy information about studies with human participants or human data. See also policy information about sex, gender (identity/presentation), and sexual orientation and race, ethnicity and racism.

Reporting on sex and gender	Not Applicable
Reporting on race, ethnicity, or other socially relevant groupings	Not Applicable
Population characteristics	Not Applicable
Recruitment	Not Applicable
Ethics oversight	Not Applicable

Note that full information on the approval of the study protocol must also be provided in the manuscript.

Field-specific reporting

Please select the one below that is the best fit for your research. If you are not sure, read the appropriate sections before making your selection.

☒ Life sciences ☐ Behavioural & social sciences ☐ Ecological, evolutionary & environmental sciences

For a reference copy of the document with all sections, see nature.com/documents/nr-reporting-summary-flat.pdf

Life sciences study design

All studies must disclose on these points even when the disclosure is negative.

Sample size	The group sample size of 6 rabbits was selected to provide enough statistical power to assess differences in serum virus neutralization assays.
Data exclusions	All data is included and shown. No data were excluded from the analyses.
Replication	Western blot analysis were performed for all groups at the same time and repeated three independent times. Negative stain electron microscopy was preformed as technical replicates from a single experiment. Serum neutralization, MSD assay, and qPCR samples were measured as technical duplicates. Flow cytometry was performed as technical replicates and representative dataset was selected from three independent experiments. All replicate attempts were successful.
Randomization	Grouping of rabbits were randomly allocated. Previous studies showed minimal differences in immunogenicity between genders so for consistency, only female rabbits were used.
Blinding	Blinding was not applied in this study to ensure appropriate immunogens were properly characterized and that the correct immunogens were delivered to the assigned animals.

Reporting for specific materials, systems and methods

We require information from authors about some types of materials, experimental systems and methods used in many studies. Here, indicate whether each material, system or method listed is relevant to your study. If you are not sure if a list item applies to your research, read the appropriate section before selecting a response.

Materials & experimental systems

n/a	Involved in the study
☐	☒ Antibodies
☐	☒ Eukaryotic cell lines
☒	☐ Palaeontology and archaeology
☐	☒ Animals and other organisms
☒	☐ Clinical data
☐	☐ Dual use research of concern
☒	☐ Plants

Methods

n/a	Involved in the study
☒	☐ ChIP-seq
☐	☒ Flow cytometry
☒	☐ MRI-based neuroimaging

Antibodies

Antibodies used

-chicken anti-Newcastle Disease Virus polyclonal antibody [ab34402] (Abcam, Cambridge, United Kingdom)
-goat anti-chicken IgY H&L- HRP conjugated secondary antibody [ab6877] (Abcam)
-rabbit anti-HIV-1 gp120 Env (Clade B, IIB) antibody [ABL#5414] (Advanced Bioscience Laboratories, Rockville, MD, USA)
-donkey anti-rabbit IgG (H+L) cross-absorbed- HRP conjugated antibody [SA1-200] (Thermo Fisher Scientific)
-mouse anti-influenza A virus (H5N1/HA1) antibody [ab135382] (Abcam)
-horse anti-mouse IgG-HRP conjugated antibody [7076S] (Cell Signaling Technology, Danvers, MA, USA)
-rabbit anti-spike (SARS-CoV2) antibody [scv2-SA-200] (eEnzyme LLC, Gaithersburg, MD, USA)
-donkey anti-rabbit IgG (H+L) cross-absorbed-HRP conjugated antibody [SA1-200] (Thermo Fisher Scientific)
-anti-Env monoclonal antibodies (PGT145, PG16, VRC01, b12, PGT151, 8ANC195, 35O22, 10E8, F105 and 447-52D) are provided by BEI Resources, Manassas, VA)
-goat anti-human IgG Fab2-phycoerythrin (PE) [109-116-097] (Jackson ImmunoResearch, West Grove, PA, USA)
-anti-Hexon (adenoviral capsid protein) antibody 8C4-allophycocyanin (APC) [NB600-413APC] (Novus Biologicals, Centennial, CO, USA)
-goat anti-rabbit SULFO-TAG™ conjugated detection antibody [R32AB-1] (Meso Scale Discovery, Rockville, MD, USA)

Validation

-All commercially purchased antibodies used in this study were validated by the supplier and source.
-anti-Env monoclonal antibodies were validated by noted DOI references:
PGT145was validated in DOI: 10.1038/nature10373
PG16 was validated in DOI: 10.1126/science.1178746
VRC01 was validated in DOI: 10.1126/science.1187659
b12 was validated in doi:10.1128/jvi.77.1.642-658.2003
PGT151 was validated in doi:10.1016/j.immuni.2014.04.009
8ANC195 was validated in doi: 10.1126/science.1207227
35O22 was validated in doi:10.1038/nature13601
10E8 was validated in doi: 10.1038/nature11544
F105 was validated in doi:10.1128/JVI.79.20.13060-13069.2005
447-52D was validated in DOI: 10.1128/JVI.66.12.7538-7542.1992

Eukaryotic cell lines

Policy information about cell lines and Sex and Gender in Research

Cell line source(s)

A549 human adenocarcinoma cells [CCL-185] (ATCC, Manassas, VA, USA)
Expi293F [A14527] (Thermo Fisher Scientific, Waltham, MA, USA)
HEK293T [CRL-3216] (ATCC, Manassas, VA, USA)
HEK293T/17 [CRL-11268] (ATCC, Manassas, VA, USA)
HEK293T/ACE [631289] (Takara Bio Inc., Kusatsu, Shiga, Japan)
HEK293A [R70507] (Thermo Fisher Scientific, Waltham, MA, USA)
TZM-bl [HRP-8129] (BEI Resources, Manassas, VA, USA)

Authentication

All cell lines used in this study were considered authenticated by the source and supplier.

Mycoplasma contamination

All cell lines used in this study were tested negative for Mycoplasma by the source and supplier.

Commonly misidentified lines
(See ICLAC register)

TZM-bl were previously designated JC53-bl (clone 13) and is a HeLa cell line.

Animals and other research organisms

Policy information about studies involving animals; ARRIVE guidelines recommended for reporting animal research, and Sex and Gender in Research

Laboratory animals	New Zealand White rabbits (Oryctolagus cuniculus) that were 6-8 weeks old were used in this study.
Wild animals	Not applicable
Reporting on sex	Female rabbits were used in this study.
Field-collected samples	No field-collected samples were used in this study.
Ethics oversight	All animal experiments were conducted according to the animal study proposal (ASP LIR21/LIRID9) approved by National Institutes of Health (NIH), National Institute of Allergy and Infectious Diseases (NIAID), Animal Care and Use Committee (ACUC) that meets all federal requirements, as defined in the Animal Welfare Act (AWA), the Public Health Service Policy (PHS), and the Humane Care and Use of Laboratory Animals in AALAC accredited facilities. This study used 6-8 weeks old female New Zealand White (NZW) rabbits (Charles Rivers Laboratories (Wilmington, MA, USA) that were co-housed, all of which were processed for terminal bleed collections under general anesthesia and euthanized by exsanguination as approved by the AVMA (American Veterinary Medical Association) and adopted by NIH-NIAID ACUC.

Note that full information on the approval of the study protocol must also be provided in the manuscript.

Plants

Seed stocks	Not applicable
Novel plant genotypes	Not applicable
Authentication	Not applicable

Flow Cytometry

Plots

Confirm that:

☒ The axis labels state the marker and fluorochrome used (e.g. CD4-FITC).

☒ The axis scales are clearly visible. Include numbers along axes only for bottom left plot of group (a 'group' is an analysis of identical markers).

☒ All plots are contour plots with outliers or pseudocolor plots.

☒ A numerical value for number of cells or percentage (with statistics) is provided.

Methodology

Sample preparation	A549 cells were transfected, harvested, stained and fixed as described in the methods section. VLPs were processed and stained as described in the methods section.
Instrument	BD FACS Aria and BD FACSymphony S6 cell sorter Flow Cytometer Instruments (Becton Dickinson)
Software	BD FACSDiva software (version 10.9.0) (Becton Dickinson)
Cell population abundance	50,000 events were recorded to detemine expression of targets on A549 cells. 10,000 events were recorded to determine expression of targets on VLPs.
Gating strategy	Expression of HIV-1 Env on A549 cells from NDV-VLPs and Ad4-Env were detected by forward (FSC) and side (SSC) scatter. Single live cells that were emitting fluorescence from anti-Env antibody confirmed expression of Env using NDV-Env plasmids. Single live cells that were emitting fluorescence from both anti-Env and anti-hexon antibodies confirmed expression of Env on Ad4-Env infected A549 cells. Voltages were adjusted to discriminate noise from single live cells expressing Env. Expression of HIV-1 Env on VLPs were detected either solely by forward (FSC) and side (SSC) scatter or using fluorescence emitted from the anti-Env antibodies. To confirm that events were indeed VLPs, the FSC and SSC thresholds and voltages were adjusted to discriminate noise from VLPs. Events were measured in PBS/BSA not containing VLPs to ensure the events in PBS/BSA were negligible compared to the number of particles detected in the samples. Cleaning with BD Detergent

Solution (BD Life Sciences, San Jose, CA) was performed as needed between each sample to ensure fewer than 50 events were detected in a tube of PBS/BSA collected over a minute at the maximum flow rate.

☒ Tick this box to confirm that a figure exemplifying the gating strategy is provided in the Supplementary Information.
